# Survival of women with clear cell and papillary serous endometrial cancer after adjuvant radiotherapy

**DOI:** 10.1186/1748-717X-9-141

**Published:** 2014-06-18

**Authors:** Robert Foerster, Robert Kluck, Harald Rief, Stefan Rieken, Juergen Debus, Katja Lindel

**Affiliations:** 1 Department of Radiation Oncology, University Hospital Heidelberg, Im Neuenheimer Feld 400, 69120 Heidelberg, Germany

**Keywords:** Radiotherapy, Endometrial cancer, Clear cell, Papillary serous

## Abstract

**Background:**

Type II (papillary serous and clear cell) endometrial carcinoma (EC) is a rare subgroup and is considered to have an unfavorable prognosis. The purpose of this retrospective analysis was to elucidate the meaning of adjuvant radiotherapy (RT) for clinical outcome and to define prognostic factors in these patients (pts).

**Methods:**

From 2004-2012 forty-two pts with type II EC underwent surgery followed by adjuvant RT at our department. Median age was 72 years. The majority were early stage carcinomas (FIGO I n = 27 [64.3%], FIGO II n = 4 [9.5%], FIGO III n = 11 [26.2%]. Seven pts (16.7%) received adjuvant chemotherapy (ChT). Pts were treated with external beam radiotherapy (EBRT) and brachytherapy (IVB) boost.

**Results:**

Five-year local recurrence free survival (LRFS), distant metastases free survival (DMFS) and overall survival (OS) were 85.4%, 78%, and 64.5% respectively. LRFS was better with lower pT stage, without lymphangiosis (L0), without haemangiosis (V0) and negative resection margins (R0). DMFS was prolonged in lymph node negatives (N0), L0, V0 and R0. OS was improved in younger pts, N0, L0, V0 and after lymphadenectomy (LNE). Multivariate analysis revealed haemangiosis (V1) as the only independent prognostic factor for OS (p = .014) and DMFS (p = .008). For LRFS pT stage remained as an independent prognostic factor (p = .028).

**Conclusions:**

Adjuvant RT with EBRT/IVB ensures adequate local control in type II EC, but control rates remain lower than in type I EC. A benefit of additional adjuvant ChT could not be demonstrated and a general omission of EBRT cannot be recommended at this point. Lymphovascular infiltration and pT stage might be the best predictive factors for a benefit from combined local and systemic treatment.

## Introduction

Uterine carcinomas with clear cell or papillary serous histology (type II) exhibit different features than endometrioid (type I) carcinomas (EC) [1]. They account for only 10% of all endometrial cancers, but analysis from historical data showed a substantially worse prognosis with frequent recurrences outside the uterus [2-4]. Due to their comparatively low incidence randomized trials on therapy and outcome of patients with theses histologic subtypes are scarce [5] and the existing retrospective studies have reported varied results regarding the effect of adjuvant radio- and chemotherapy [6,7]. Another constraint of previously published studies is that they included heterogeneously treated patients over a time frame of more than 20 years [8-10] or that data were extracted from prospective studies which included only a small percentage of type II EC [6].

This study was planned to investigate the prognosis and survival of women who were treated in recent years with a combined approach of local and systemic therapy.

## Methods

Screening our patient database we analyzed 410 women who underwent adjuvant radiotherapy (RT) for EC at the Department of Radiation Oncology at the University Hospital Heidelberg in Germany between 2004 and 2012. Among those, 42 cases presented carcinomas with clear cell, papillary serous or mixed histology and thus were included in this retrospective analysis.

All patients were operated with simple hysterectomy, bilateral salpingo-oophorectomy and omental biopsy. In 15 (35.7%) women only pelvic and in 17 (40.5%) pelvic and paraaortic lymph node dissection was performed. RT consisted of external beam radiotherapy (EBRT) in 1.8-2.0 Gy fractions to a cumulative dose of 40-54 Gy (median 45 Gy) and/or HDR intravaginal brachytherapy (IVB) in 5.0 Gy fractions to a cumulative dose of 10-20 Gy (median 10 Gy).

Follow-up was documented and detailed information was gathered on stage, grading, resection status, lymph and blood vessel involvement as well as on prescription of chemotherapy (ChT). For FIGO staging the 2009 classification was used and patients were reclassified if necessary [11].

Local recurrence free survival (LRFS) was calculated as the time between first diagnosis and occurrence of first local recurrence; with local recurrence being defined as a relapse in the irradiation field. Distant metastases free survival (DMFS) was calculated from day of first diagnosis until day of distant relapse. Disease free survival (DFS) was considered to be the time from first diagnosis until local or distant recurrence. Overall survival (OS) was calculated from date of first diagnosis until death from any cause.

Survival was calculated according to Kaplan and Meier and the log-rank test was used for univariate statistical evaluation. Cox proportional regression model was used for multivariate analysis. A p-value ≤ 0.05 was considered statistically significant.

Statistical analysis was performed with SPSS 22.0 for Windows.

Our research was carried out in compliance with the Helsinki Declaration.

## Results

### Patients’ and tumor characteristics

Table 1 summarizes patients’ characteristics.

**Table 1:** Patients’ characteristics

	**n**	**%**
**Histology N = 42**		
Clear cell	13	31.0%
Papillary serous	20	47.6%
Mixed	9	21.4%
**FIGO stage N = 42**		
IA	23	54.8%
IB	4	9.5%
II	4	9.5%
IIIA	1	2.4%
IIIB	1	2.4%
IIIC	9	21.4%
**Local tumors spread N = 42**		
pT1a	26	61.9%
pT1b	6	14.3%
pT2	5	11.9%
pT3a	4	9.5%
pT3b	1	2.4%
**Lymph node status N = 35**		
pN0	26	74.3%
pN1	9	25.7%
**Lymphangiosis N = 41**		
L0	33	80.5%
L1	8	19.5%
**Haemangiosis N = 41**		
V0	34	82.9%
V1	7	17.1%
**Resection status N = 42**		
R0	36	85.7%
R1	6	14.3%

Mean age at diagnosis was 69.9 years (range: 41-89 years). Thirteen women (31%) had clear cell, 20 (47.6%) had papillary serous and 9 (21.4%) had mixed histology carcinomas.

FIGO stages were distributed as follows: stage I 64.3%, stage II 9.5% and stage III 26.2%. None were stage IV. Nine women (25.7%) had positive lymph nodes. Pelvic lymph nodes were resected in 15 patients (35.7%) and in 17 (40.5%) additionally paraaortic lymph nodes were resected. In 19.5% (n = 8) the tumor had infiltrated the lymph and in 17.1% (n = 7) the blood vessels. Positive microscopic resection margins (R1) were found in 14.3% (n = 6) of patients.

Seven patients (16.7%) were offered adjuvant ChT with carboplatin and paclitaxel before or after RT. Except for one patient with a stage II carcinoma all patients who received chemotherapy were diagnosed with stage III disease. Treatment is shown in Table 2.

**Table 2:** Treatment

	**n**	**%**
**Surgery N = 42**		
Hysterectomy and bilateral salpingo-oophorectomy	42	100%
Lymphadenectomy		
Pelvic	15	35.7%
Pelvic and paraaortic	17	40.5%
None	10	23.8%
**Adjuvant chomotherapy N = 42**		
Carboplatin/paclitaxel	7	16.7%
None	35	83.3%
**Adjuvant radiotherapy N = 42**		
Intravaginal HDR brachytherapy alone	5	11.9%
Intravaginal HDR brachytherapy and external beam radiotherapy	37	88.1%

### Survival analysis

**Figure 1:**
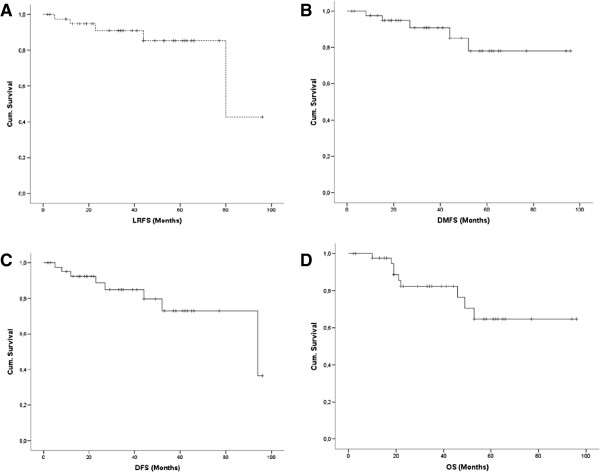
Kaplan-Meier survival curves for (A) LRFS, (B) DMFS, (C) DFS and (D) OS.

Mean follow-up was 36.8 months. Nine patients (24.1%) died during follow-up, five (11.9%) developed a local recurrence and five (11.9%) developed distant metastases. Statistical 5 year LRFS, DMFS, DFS and OS were 85.4%, 78%, 73% and 64.5% respectively (Figure 1). Distant relapses occurred in lungs (n = 3), skin (n = 1), abdominal wall (n = 1) and inguinal lymph nodes (n = 1).

### Univariate analysis

Local control was better with lower FIGO stage (p = .031), lower pT stage (p = .001), negative resection margins (p = .037), when lymph or blood vessels were not infiltrated (p = .011; p = .015) and no ChT was applied (p = .048).

Regarding DMFS higher FIGO stage (p = .028), positive lymph nodes (p = .001), positive resection margins (p = .048) as well as lymph and blood vessel infiltration (p = .001; p = .001) were negative prognostic factors.

DFS was prolonged with lower pT stage (p = .009), negative lymph node status (p = .006), no lymph or blood vessel infiltration (p = .001; p = .001), negative resection margins (p = .013) and without ChT (p = .025).

OS was significantly longer in younger patients (p = .028) without lymph node metastases (p = .026), when lymphadenectomy was performed (p = .004) and when neither lymph nor blood vessels were infiltrated by the tumor (p = .016; p = .023).

### Multivariate analysis

**Figure 2:**
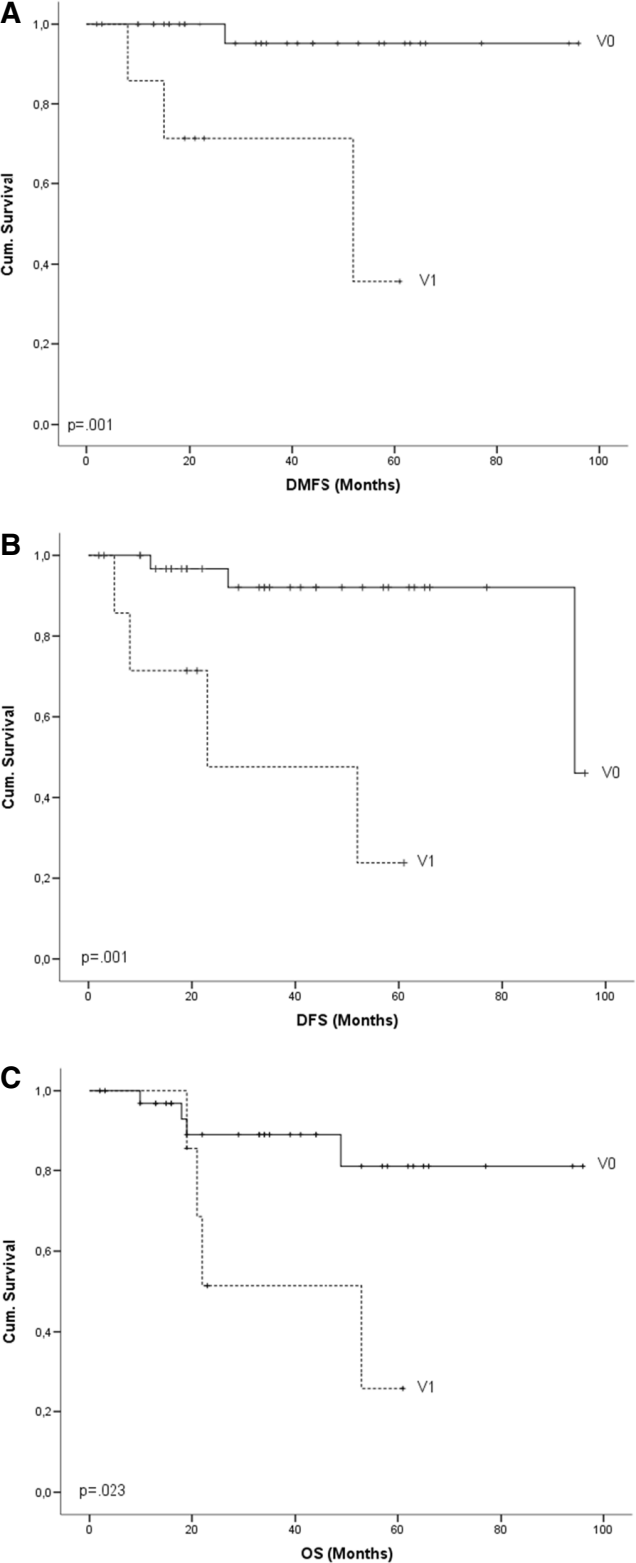
Kaplan-Meier survival curves for haemangiosis and (A) DMFS, (B) DFS, (C) OS.

**Figure 3:**
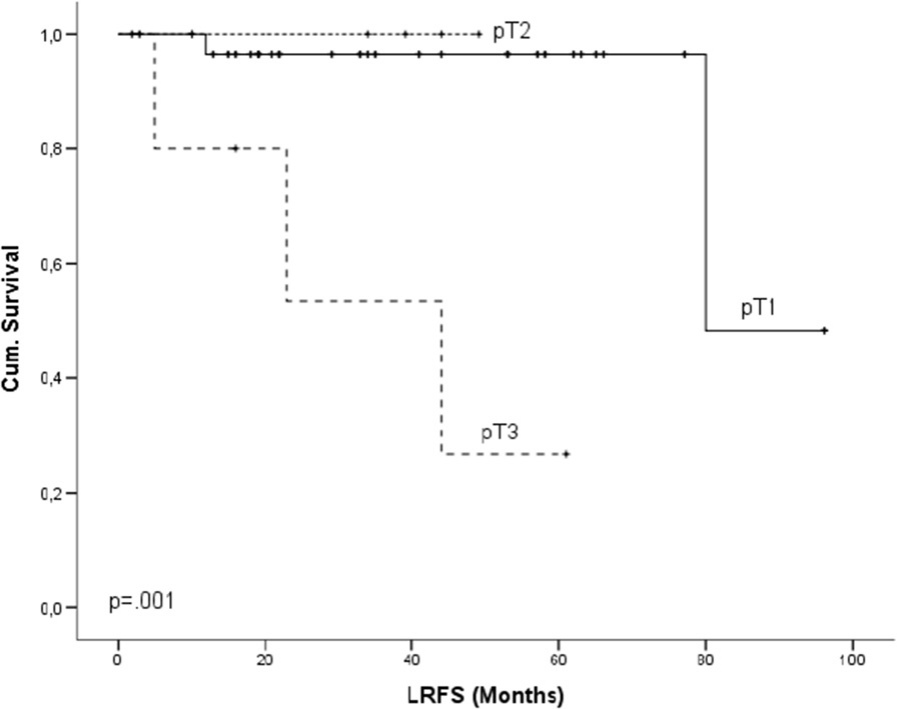
Kaplan-Meier survival curves for pT stage and LRFS.

Cox regression model revealed haemangiosis (Figure 2) as the only independent prognostic factor for OS (p = .014), DMFS (p = .008) and DFS (p = .007). For LRFS pT stage (Figure 3) was calculated to be the only independent prognostic factor (p = .028).

## Discussion

In agreement with other research groups we report a majority of papillary serous carcinomas among type II EC [4,9,12].

In recent years efforts have been made to reduce adjuvant treatment to IVB in combination with systemic ChT. While promising results have been published in early stage carcinomas [13], our results do not support this trend for type II EC in general. Although almost two thirds of the patients in our analysis had stage I carcinomas, survival was generally worse than in patients with type I EC and our results regarding OS and DFS coincide with other reports [4,5,8-10,14-16].

Local control was adequate with 85.4% LRFS after 5-years. This supports previous findings that adjuvant radiotherapy is capable of ensuring a good local control [10,17]. However, it was far from control rates in women with type I carcinomas; despite that most of our patients (88.1%) underwent combined treatment with EBRT and IVB. Omission of one treatment modality cannot be recommended at this point.

In our analysis only 17% of patients received adjuvant ChT and adding ChT to RT could potentially improve outcome [6,10,14,18,19]. However, the overall survival benefit from carboplatin/paclitaxel ChT was poor in our analysis and the efficiency of the established taxol/platinum based ChT regimens in type II EC may be questioned. Regardless of the selection bias of treating predominantly stage III patients with adjuvant radiochemotherapy (RChT), which resulted in a significantly worsened outcome after ChT, there was no statistically significant survival difference between the sole RT and the RChT groups. Although numbers are small, this points toward the necessity of new agents and further investigation of molecular targeted therapies such as inhibitors of vascular endothelial growth factor (VEGF) and mammalian target of rapamycin (mTOR) or tyrosin kinase inhibitors (TKI) [20].

Regarding primary surgical therapy, we found that the conduction of lymphadenectomy was associated with prolonged overall survival in our study, stressing the importance of comprehensive surgical staging [21,22]. Performance of selective surgical staging, as it is standard for endometrioid uterine carcinoma, cannot be recommended [23,24].

In our analysis lymphangiosis and haemangiosis were prognostic factors of LRFS, DMFS, DFS and OS in univariate analysis and blood vessel infiltration remained the only independent prognostic factor in multivariate analysis of DMFS, DFS and OS. Lymphovascular invasion has previously been associated with prolonged survival [10,12,25-27]. Additionally we found pT stage to be the only independent prognostic factor of prolonged LRFS. Lymphovascular invasion and extend of local tumor spread may therefore be the best predictors of a benefit from combined local and systemic treatment.

Future efforts should be directed towards multi-institutional randomized trials to enable a better understanding of this highly malignant disease’s therapy requirements.

## Conclusion

Adjuvant RT with EBRT/IVB ensures adequate local control in type II EC, but control rates remain lower than in type I EC. A benefit of additional adjuvant ChT could not be demonstrated and omission of EBRT, as conducted in early stage type I carcinoma, cannot be recommended at this point. Lymphovascular invasion pT stage might be the best predictive factors for a benefit from combined local and systemic treatment.

## Competing interests

The authors declare that they do not have any competing interests.

## Authors’ contributions

RF and KL developed and planned this retrospective study. RK and RF were responsible for data collection and statistical analysis. All authors read and approved the final manuscript.

## References

[B1] Mendivil A Schuler KM Gehrig PA Non-endometrioid adenocarcinoma of the uterine corpus: a review of selected histological subtypes Cancer Control 2009 16 46 52 19078929 10.1177/107327480901600107

[B2] Boruta DM Gehrig PA Fader AN Olawaiye AB Management of women with uterine papillary serous cancer: a Society of Gynecologic Oncology (SGO) review Gynecol Oncol 2009 115 142 153 10.1016/j.ygyno.2009.06.011 19592079

[B3] Greggi S Mangili G Scaffa C Scala F Losito S Iodice F Pisano C Montoli S Vigano R Pirozzi G Giannarelli D Uterine papillary serous, clear cell, and poorly differentiated endometrioid carcinomas: a comparative study Int J Gynecol Cancer 2011 21 661 667 10.1097/IGC.0b013e3182150c89 21412164

[B4] Hamilton CA Cheung MK Osann K Chen L Teng NN Longacre TA Powell MA Hendrickson MR Kapp DS Chan JK Uterine papillary serous and clear cell carcinomas predict for poorer survival compared to grade 3 endometrioid corpus cancers Br J Cancer 2006 94 642 646 16495918 10.1038/sj.bjc.6603012 PMC2361201

[B5] Fields AL Einstein MH Novetsky AP Gebb J Goldberg GL Pilot phase II trial of radiation “sandwiched” between combination paclitaxel/platinum chemotherapy in patients with uterine papillary serous carcinoma (UPSC) Gynecol Oncol 2008 108 201 206 10.1016/j.ygyno.2007.09.025 17997145

[B6] Johnson N Bryant A Miles T Hogberg T Cornes P Adjuvant chemotherapy for endometrial cancer after hysterectomy Cochrane Database Syst Rev 2011 CD003175 21975736 10.1002/14651858.CD003175.pub2 PMC4164379

[B7] Morneau M Foster W Lalancette M Van Nguyen-Huynh T Renaud MC Samouelian V Letarte N Almanric K Boily G Bouchard P Boulanger J Cournoyer G Couture F Gervais N Goulet S Guay MP Kavanagh M Lemieux J Lesperance B Letarte N Morneau M Ouellet JF Pineau G Rajan R Roy I Samson B Sideris L Vincent F Adjuvant treatment for endometrial cancer: literature review and recommendations by the Comite de l'evolution des pratiques en oncologie (CEPO) Gynecol Oncol 2013 131 231 240 10.1016/j.ygyno.2013.07.084 23872191

[B8] Kim HJ Kim TJ Lee YY Choi CH Lee JW Bae DS Kim BG A comparison of uterine papillary serous, clear cell carcinomas, and grade 3 endometrioid corpus cancers using 2009 FIGO staging system J Gynecol Oncol 2013 24 120 127 10.3802/jgo.2013.24.2.120 23653828 PMC3644687

[B9] Scarfone G Secomandi R Parazzini F Vigano R Mangili G Frigerio L Villa A Tateo S Ricci E Bolis G Clear cell and papillary serous endometrial carcinomas: survival in a series of 128 cases Arch Gynecol Obstet 2013 287 351 356 10.1007/s00404-012-2586-x 23100038

[B10] Viswanathan AN Macklin EA Berkowitz R Matulonis U The importance of chemotherapy and radiation in uterine papillary serous carcinoma Gynecol Oncol 2011 123 542 547 10.1016/j.ygyno.2011.09.005 21963091

[B11] Pecorelli S Revised FIGO staging for carcinoma of the vulva, cervix, and endometrium Int J Gynaecol Obstet 2009 105 103 104 10.1016/j.ijgo.2009.02.012 19367689

[B12] Craighead PS Sait K Stuart GC Arthur K Nation J Duggan M Guo D Management of aggressive histologic variants of endometrial carcinoma at the Tom Baker Cancer Centre between 1984 and 1994 Gynecol Oncol 2000 77 248 253 10.1006/gyno.2000.5746 10785473

[B13] Kiess AP Damast S Makker V Kollmeier MA Gardner GJ Aghajanian C Abu-Rustum NR Barakat RR Alektiar KM Five-year outcomes of adjuvant carboplatin/paclitaxel chemotherapy and intravaginal radiation for stage I-II papillary serous endometrial cancer Gynecol Oncol 2012 127 321 325 10.1016/j.ygyno.2012.07.112 22850412

[B14] Goldberg H Miller RC Abdah-Bortnyak R Steiner M Yildiz F Meirovitz A Villa S Poortmans PM Azria D Zidan J Ozsahin M Abacioglu U Gold DG Amit A Lavie O Atahan IL Kuten A Outcome after combined modality treatment for uterine papillary serous carcinoma: a study by the Rare Cancer Network (RCN) Gynecol Oncol 2008 108 298 305 10.1016/j.ygyno.2007.10.037 18096209

[B15] Yechieli R Rasool N Robbins JR Cogan CM Elshaikh MA Adjuvant radiation therapy for patients with type II endometrial carcinoma: impact on tumor recurrence and survival Int J Gynecol Cancer 2013 23 763 768 10.1097/IGC.0b013e31828b15cb 23485931

[B16] Creutzberg CL van Putten WL Koper PC Lybeert ML Jobsen JJ Warlam-Rodenhuis CC De Winter KA Lutgens LC van den Bergh AC Steen-Banasik E Beerman H van LM Surgery and postoperative radiotherapy versus surgery alone for patients with stage-1 endometrial carcinoma: multicentre randomised trial. PORTEC Study Group. Post Operative Radiation Therapy in Endometrial Carcinoma Lancet 2000 355 1404 1411 10.1016/S0140-6736(00)02139-5 10791524

[B17] Murphy KT Rotmensch J Yamada SD Mundt AJ Outcome and patterns of failure in pathologic stages I-IV clear-cell carcinoma of the endometrium: implications for adjuvant radiation therapy Int J Radiat Oncol Biol Phys 2003 55 1272 1276 10.1016/S0360-3016(02)04404-8 12654437

[B18] Fader AN Drake RD O'Malley DM Gibbons HE Huh WK Havrilesky LJ Gehrig PA Tuller E Axtell AE Zanotti KM Platinum/taxane-based chemotherapy with or without radiation therapy favorably impacts survival outcomes in stage I uterine papillary serous carcinoma Cancer 2009 115 2119 2127 10.1002/cncr.24247 19306417

[B19] Hogberg T Signorelli M de Oliveira CF Fossati R Lissoni AA Sorbe B Andersson H Grenman S Lundgren C Rosenberg P Boman K Tholander B Scambia G Reed N Cormio G Tognon G Clarke J Sawicki T Zola P Kristensen G Sequential adjuvant chemotherapy and radiotherapy in endometrial cancer–results from two randomised studies Eur J Cancer 2010 46 2422 2431 10.1016/j.ejca.2010.06.002 20619634 PMC3552301

[B20] Thanapprapasr D Cheewakriangkrai C Likittanasombut P Thanapprapasr K Mutch DG Targeted endometrial cancer therapy as a future prospect Womens Health (Lond Engl) 2013 9 189 199 10.2217/whe.13.4 23477324

[B21] Chan JK Cheung MK Huh WK Osann K Husain A Teng NN Kapp DS Kapp DS Therapeutic role of lymph node resection in endometrioid corpus cancer: a study of 12,333 patients Cancer 2006 107 1823 1830 10.1002/cncr.22185 16977653

[B22] Fader AN Boruta D Olawaiye AB Gehrig PA Uterine papillary serous carcinoma: epidemiology, pathogenesis and management Curr Opin Obstet Gynecol 2010 22 21 29 10.1097/GCO.0b013e328334d8a3 19952744

[B23] Bristow RE Asrari F Trimble EL Montz FJ Extended surgical staging for uterine papillary serous carcinoma: survival outcome of locoregional (Stage I-III) disease Gynecol Oncol 2001 81 279 286 10.1006/gyno.2001.6159 11330963

[B24] del Carmen MG Birrer M Schorge JO Uterine papillary serous cancer: a review of the literature Gynecol Oncol 2012 127 651 661 10.1016/j.ygyno.2012.09.012 23000148

[B25] Vance S Yechieli R Cogan C Hanna R Munkarah A Elshaikh MA The prognostic significance of age in surgically staged patients with Type II endometrial carcinoma Gynecol Oncol 2012 126 16 19 10.1016/j.ygyno.2012.04.011 22507535

[B26] Weber SK Sauerwald A Polcher M Braun M Debald M Serce NB Kuhn W Brunagel-Walgenbach G Rudlowski C Detection of lymphovascular invasion by D2-40 (podoplanin) immunoexpression in endometrial cancer Int J Gynecol Cancer 2012 22 1442 1448 10.1097/IGC.0b013e318269139b 22964524

[B27] Weinberg LE Kunos CA Zanotti KM Lymphovascular Space Invasion (LVSI) Is an Isolated Poor Prognostic Factor for Recurrence and Survival Among Women With Intermediate- to High-Risk Early-Stage Endometrioid Endometrial Cancer Int J Gynecol Cancer 2013 23 1438 1445 10.1097/IGC.0b013e3182a16c93 24257558

